# The Correct Nutritional Intake in the Prevention and Treatment of Skin Lesions in Patients With Spinal Cord Injury

**DOI:** 10.14740/jocmr6399

**Published:** 2026-03-26

**Authors:** Alessandra Areni, William Capeci, Alessandro Corsi, Giulio Del Popolo, Luisa De Palma, Laura Pelizzari, Primo Vercilli

**Affiliations:** a AUSL Bologna, Bologna, Italy; b Spinal Cord Unit, Department of Neurogical Science, University Hospital of Marche, Ancona, Italy; c B. Braun SpA, Milan, Italy; d Spinal Unit, Department of Neuroscience and Translational Medicine, Azienda Ospedaliero-Universitaria Policlinico, Bari, Italy; e Department of Neuro-Urology & Spinal Unit, Azienda Ospedaliera-Universitaria Careggi, Firenze, Italy; f Spinal Unit and Neurorehabilitation, Department of Rehabilitation Medicine, AUSL Piacenza, Piacenza, Italy; g Ambulatorio di Nutrizione, Poliambulatorio Chiros, Forli, Italy

**Keywords:** Spinal cord injury, Pressure injuries, Nutrition therapy, Wound healing, Gut microbiota, Inflammation, Nutrient supplementation

## Abstract

**Background:**

Pressure injuries (PIs) are a serious and highly prevalent complication in individuals with spinal cord injury (SCI), negatively impacting quality of life, rehabilitation, and healthcare costs. Given their multifactorial etiology, a multidisciplinary approach that includes nutritional interventions is essential. This review provides expert consensus on the role of nutrition in PI prevention and management in SCI patients.

**Methods:**

A panel of clinicians, including experts in wound care, nutrition, urology, internal medicine, and rehabilitation, assessed current practices and proposed evidence-based dietary recommendations. A narrative literature review supported the consensus process.

**Results:**

PI risk screening through SCI-specific tools (e.g., Braden scale) and shared decision-making were supported. Nutritional recommendations include aligning energy intake to reduced total daily energy expenditure, highly digestable protein rich in essential amino acids, omega-3 fatty acids, and micronutrients, with adequate hydration. Dietary patterns should be anti-inflammatory, rich in fiber, fruits/vegetables, legumes, fish, and fermented foods. Also, they should modulate gut microbiota and reduce advanced glycation end products through food choices and cooking methods. Practical tools, the Food Suitability Map (traffic-light guidance) and food diary, were proposed to support education, adherence, and self-management. Implementation guidance is provided for acute, chronic, community, and readmission/surgical settings.

**Conclusions:**

This review stresses the urgent need for standardized, SCI-specific nutritional protocols and enhanced interdisciplinary collaboration. Nutrition should be integrated into routine PI care to improve patient outcomes and reduce healthcare burden. Future research should explore nutraceuticals, refine existing protocols, assess long-term impacts of dietary strategies, and strengthen implementation in clinical practice.

## Introduction

Spinal cord injury (SCI) is defined as any damage to the spinal cord resulting in partial or complete loss of motor, sensory, and autonomic function below the level of injury. Higher-level injuries, particularly cervical SCIs, lead to tetraplegia and profound immobility, severely limiting functional independence. Thoracic or lumbar SCIs typically result in paraplegia, with preserved upper limb function but substantial limitations in lower-body mobility [1]. SCI leads to complications that significantly impact quality of life (QoL), including respiratory dysfunction, neurogenic bowel and bladder, osteoporosis, pressure injuries (PIs), and alterations in metabolism that lead to changes in body composition such as sarcopenia and obesity [2–6]. Tetraplegia profoundly increases the risk of malnutrition due to reduced muscle mass, impaired gastrointestinal function, and greater metabolic alterations and paraplegia contributes to reduced physical activity and altered nutrient requirements [7]. Neurogenic obesity occurs due to low metabolic rate and total daily energy expenditure (TDEE) [8] and increases cardiometabolic risk [9]. Also, gut microbiome changes that occur in SCI contribute to chronic inflammation and metabolic disorders [10]. Early after SCI, weight loss may occur due to increased metabolic demand from trauma [11] or undernutrition driven by infections, dysphagia, hormonal changes, wounds, and psychological stress [12].

PIs are localized skin or tissue injury from pressure or shear [13] and, despite being preventable, 20–50% of acute SCI patients develop PIs in the hospital [14]. PI annual incidence in SCI patients ranges from 25.3% to 41.0% [15] and lifetime risk reaches 85.0–95.0% [16]. Malnutrition, which affects 86% of SCI patients with PIs [17, 18], is a major risk factor for PI severity and delayed healing [19]. PIs cause infections, impair rehab, reduce well-being, extend hospital stays, and raise mortality [20–22]. They impose high healthcare costs ($4,745/month) [23], along with indirect costs and caregiver burden [24]. PIs may represent 25% of SCI care costs, with treatment more expensive than prevention [25, 26].

This review explores PI prevention and treatment strategies across care settings, emphasizing the role of targeted nutritional interventions in SCI.

## Methods

A multidisciplinary panel, including a wound care physician, urologist, physician nutrition specialist, two internal medicine specialists, and two physical medicine and rehabilitation physicians, met in July 2024 to address PI management in SCI patients in Italy. The group evaluated PI risk and reviewed prevention and treatment strategies, with a focus on identifying nutritional deficiencies and proposing dietary interventions to support wound healing. This review presents consensus-based recommendations (≥ 70% agreement). A narrative literature review was also conducted to support the discussions. Articles were selected based on their relevance following targeted searches in PubMed. The search strategy included combinations of the following keywords and Boolean operators: “spinal cord injury” AND “pressure injury” OR “pressure ulcer” OR “pressure sores”; “spinal cord injury” AND “risk factors” OR “mobility limitations” OR “skin integrity” OR “autonomic dysreflexia”; “spinal cord injury” AND “risk assessment” OR “risk assessment tools” OR “Braden Scale” OR “SCIPUS” OR “SCIPUS-A” OR “LPD Scale”; “spinal cord injuries” AND “nutrition assessment” OR “nutritional status”; “spinal cord injury” AND “change in body composition” OR “malnutrition” OR “undernutrition” OR “dietary supplements”; “spinal cord injury” AND “energy expenditure”; “anti-inflammatory diets”; “anti-inflammatory nutrients”; “Mediterranean diet” AND “inflammation”; “spinal cord injury” AND “gastrointestinal microbiome” OR dysbiosis; “spinal cord injury” AND “multidisciplinary”.

## Results

### PI preventive strategies: a multi-faceted approach

Effective management of SCI patients should prioritize early PI risk assessment, the timely implementation of preventive strategies (e.g., specialized pressure redistribution devices) and early detection [25]. Preventing PIs requires a lifelong commitment, emphasizing the need for continuous education for patients and structured training programs for healthcare professionals (HCPs) [26–28]. As growing evidence underscores the critical role of nutrition in wound healing, targeted dietary interventions might support the prevention of PIs and tissue repair while reducing complications [29].

#### Comprehensive risk assessment, predictive tools for PIs, and PI management

Previously published data have categorized PI risk factors into intrinsic and extrinsic [30–32]. Building on this evidence and their clinical experience, the authors have drawn attention to the most relevant elements that increase vulnerability to PI development and delay wound healing (Table 1).

**Table 1:** Risk Factors for PIs in SCI Patients

Risk factor	Description
Intrinsic factors	
Impaired sensation	Loss of sensory perception prevents pain or discomfort awareness, causing prolonged pressure on tissues.
Reduced mobility	Limited or absent ability to reposition independently increases pressure exposure.
Muscle atrophy	Loss of muscle mass reduces padding on bony prominences, increasing PI risk.
Autonomic dysregulation	Poor circulation and thermoregulation impair skin health and wound healing.
Malnutrition	Protein and micronutrient deficiencies weaken skin integrity and delay healing.
Incontinence (urinary/fecal)	Moisture and bacteria increase the risk of skin breakdown and infection.
Spasticity or contractures	Abnormal muscle tone can cause friction, shear, and prolonged pressure on certain areas.
Comorbidities (e.g., diabetes, cardiovascular disease)	Conditions affecting circulation, immune function, or healing increase susceptibility.
Advanced age	Aging leads to thinner and less elastic skin, reduced tissue perfusion, and delayed wound healing, increasing susceptibility to PIs. Slower cell turnover and reduced blood supply impair skin integrity and repair processes. Aging-related comorbidities (e.g., diabetes, vascular disease) further increase the risk.
Extrinsic factors	
Prolonged pressure on bony areas	Prolonged sitting or lying down without position changes causes ischemia and tissue damage.
Shear and friction	Skin movement against surfaces can disrupt blood flow and cause injury.
Excess moisture	Sweating or incontinence weakens skin integrity, making it more prone to breakdown.
Improper support surfaces	Inadequate mattresses, cushions, or positioning devices fail to distribute pressure effectively.
Poor nursing or caregiver support	Inadequate pressure relief strategies, skin checks, and hygiene contribute to injury risk.

PI: pressure injury.

The authors have also emphasized the need for a collaborative approach between patients and the multidisciplinary team, including physical medicine and rehabilitation physicians, wound-care specialists, neurologists, internal medicine specialists, urologists, physician nutrition specialists, and nurses [33]. This model aims at improving prevention strategies and outcomes through shared decision-making. Also, access to relevant health information enhances adherence to preventive measures and supports effective self-care. Given patient variability, especially in acute phases, regular reassessment is needed. In community settings, where limited time and resources constrain HCPs to episodic contact, patients and their families are increasingly expected to manage their own care [34].

The authors reviewed the reliability of tools used to predict PI risk in SCI patients (Table 2 [31, 33, 35–39]).

**Table 2:** Existing Evidence on the Use of Risk Assessment Tools for PI Prediction in SCI Populations

Tool	Purpose/context	Strengths	Limitations	Notes
Braden Scale	Commonly used in acute and readmission SCI settings	Good predictive value in SCI outpatients [35] Broad predictive scope	Effectiveness in home care is uncertain [36] No significant benefit vs. clinical judgment [37] Insufficient nutritional assessment Relies on subjective inputs	Remains a reference tool, especially in acute settings
SCIPUS	Predictive tool for SCI-specific risks	Includes SCI-specific risks, such as autonomic dysreflexia [38]	Modest predictive accuracy Not validated in Italy	Combining Braden ≤ 12 and SCIPUS ≥ 9 may enhance HAPI prediction [31, 38]
SCIPUS-A	Adapted version of SCIPUS	Targets SCI-specific risk assessment	Modest accuracy Remains unvalidated in Italy	Similar predictive limitations to SCIPUS
LPD Scale	Designed for chronic/home/community SCI management	Includes unique home care factors, such as caregiver support and hygiene [39]	Not suitable for acute settings Lacks nutritional/clinical input	Developed specifically for home care; may outperform Braden in this context
Combined Use	Braden + SCIPUS + context-specific risk factors	Enhances HAPI prediction by integrating general and SCI-specific risks [31, 38]	No unified validation Requires regular reassessment due to patient variability	Encouraged to apply shared decision-making models in PI risk assessment [33]

HAPI: hospital-acquired pressur injury; PI: pressure injury; SCI: spinal cord injury.

Four key themes have been identified regarding patient perceptions of PI risk and the communication of risk between nurse and patient in the home setting: 1) PI awareness; 2) importance of repositioning; 3) healthy eating; and 4) risk interpretation [40]. Accordingly, the authors stressed the need to educate HCPs in PI prevention, detection, and management, possibly through master’s degree programs in SCI-related PI care.

**Figure 1:**
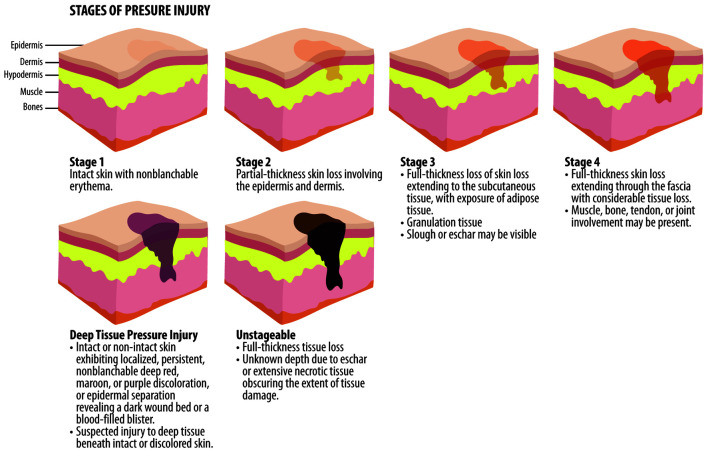
Stages of PIs based on EPUAP classification. EPUAP: European Pressure Ulcer Advisory Panel; PIs: pressure injuries.

The authors underlined the importance of an assessment by wound-care expert physicians and/or nurses trained in wound care to classify the PI based on staging (Fig. 1) [41].

The importance of multidisciplinary pre- and postoperative care and patient education has been highlighted in prior research. This strategy is based on the collaboration between plastic surgeons, internal medicine specialists, and physical medicine and rehabilitation physicians [30]. Key clinical domains involved in the holistic management of patients with PIs, emphasizing a multidisciplinary approach, are outlined in Table 3 [30, 42–51].

**Table 3:** Comprehensive Multidisciplinary Assessment Domains for Patients With Chronic PIs

Category	Details	References
Multidisciplinary care	Collaboration between plastic surgeons, internal medicine specialists, and physical medicine and rehabilitation physicians; emphasized for pre- and postoperative care	[30]
Wound assessment and microbial load measurement	Assess bleeding, exudate type, odor, color. Levine technique or culture biopsy for microbial load; swab analysis generally ineffective	[42]
Infectious disease management	Infectious disease specialists guide antibiotic therapy in suspected osteomyelitis; MRI preferred over CT for spongiosa edema	–
First-line treatment	Wound care, offloading, nutritional support	–
Surgical intervention	Indicated for advanced or non-healing PIs; plastic surgeon required	[30]
Surgical techniques	Debridement with direct closure or vascularized tissue flaps (myocutaneous, fasciocutaneous, free flaps with skin grafting)	[43]
Routine clinical assessments	CBC, CRP, ESR, renal function tests (BUN, creatinine), electrolytes, vitamins, albumin, lipid profiles for nutritional/metabolic evaluation	[44]
Malnutrition indicators	Pre-albumin < 11 mg/dL + CRP < 15 mg/L indicates malnutrition while excluding inflammation/infection	[45]
Energy and anthropometric assessment	Indirect calorimetry for resting and total daily energy expenditure (TDEE); assess height, weight, BMI	[46]
Neurogenic obesity	BMI ≥ 22 kg/m^2^ is often linked with prediabetes, hypertension	[47]
Cachexia and anorexia evaluation	Assess free fat mass; rule out psychogenic and organic causes	–
Hydration and sepsis monitoring	Monitor hydration and procalcitonin levels	[48]
Gut microbiota	Dysbiosis is common; pathogenic, pro-inflammatory bacteria ↑; SCFA-producing bacteria ↓; affects gut barrier and inflammation	[49]
QoL evaluation	Use SCI-QOL and WHOQOL tools; assess physical, psychological, and social domains, and independence for patient-centered rehabilitation	[50, 51]

BMI: body mass index; BUN: blood urea nitrogen; CBC: complete blood count; CRP: C-reactive protein; CT: computed tomography; ESR: erythrocyte sedimentation rate; MRI: magnetic resonance imaging; PI: pressure injury; QoL: quality of life; SCFA: short-chain fatty acids; SCI-QOL: spinal cord injury–quality of life; TDEE: total daily energy expenditure; WHOQOL: World Health Organization Quality of Life; TDEE: total energy expenditure.

### The role of nutrition in wound healing of PIs in SCI patients

Although precise nutrient needs for SCI patients remain unclear, high-protein oral supplements have been shown to reduce PI incidence by 25% in at-risk individuals. Energy, protein, arginine, glutamine, omega-3 fatty acids, and key micronutrients, such as vitamins A, B, C, zinc, and iron, support collagen synthesis, inflammation control, and tissue repair [52, 53]. Essential amino acids aid in nitrogen balance and promote fibroblast proliferation, angiogenesis, and immune function. Adequate hydration maintains skin integrity, cellular metabolism and blood flow to injured tissue, preventing further skin damage and delayed wound healing [52, 53]. Malnutrition compromises collagen formation and immune response, worsening PI outcomes [54, 55]. Chronic wounds, such as PIs, sustain prolonged inflammation, with low growth factor levels and microbial contamination. Hypermetabolism and a catabolic state further raise protein needs and TDEE [56]. In this context, the intake of protein sources highly digestable and containing essential aminoacids is crucial [57, 58].

#### The nutritional needs of SCI patients and SCI patients with PIs

Published data reports that TDEE ranges from 2,030 to 3,344 kcal/day in the acute SCI phase, and from 1,332 to 2,834 kcal/day in chronic SCI. TDEE is reduced by up to 54% in individuals with tetraplegia and around 20% in those with paraplegia [9, 59]. Despite generally high protein intake, SCI patients may lack essential amino acids, which are vital for protein synthesis and tissue repair, particularly important in the context of PIs [60, 61]. Moreover, PI development rapidly depletes protein reserves, mainly skeletal muscle, accelerating malnutrition [62]. Accordingly, to identify patients at nutritional risk, the Spinal Nutrition Screening Tool (SNST), a validated, SCI-specific screening instrument, assesses skin integrity alongside several SCI-related and general clinical factors. These include age, weight history, appetite, injury level, comorbidities (e.g., need for artificial ventilation or nutrition), use of supplements, modified-texture diets, and the individual’s ability to eat. Each item is scored from 0 to 5, and the total score indicates the malnutrition risk category: 0–10 = low risk, 11–15 = moderate risk, and > 15 = high risk [63].

Guidelines recommend in SCI patients without PIs or infection an intake of 0.8–1.0 g/kg/day of protein to maintain protein status [62] and limiting saturated fat to 5–6% of total energy intake [64]. Carbohydrates should constitute 45–65% of daily energy intake, preferably from complex, fiber-rich, low-glycemic sources. A fiber intake of 25–30 g/day is advised to support metabolism and bowel function, and prevent neurogenic bowel dysfunction [62]. Although guidelines exist [9, 64, 65], there is an urgent need for innovative nutrition protocols tailored to SCI patients at risk of or presenting with PIs [66].

#### Emerging perspectives on diet in SCI

Tailored nutrition is essential for preventing PIs and promoting wound healing and should reflect specific clinical needs, such as metabolic syndrome or post-surgical recovery. Annual assessments and education by registered dietitians should be routine. Supplements may support outcomes but must follow individualized plans based on comprehensive evaluations addressing inflammation, oxidative stress, and immunity.

Diets should balance macronutrients to meet reduced TDEE and prioritize nutrient-dense, low-energy foods [67]. Given SCI-related immune dysfunction and inflammation, and comorbidities (e.g., diabetes and hypertension) [68], nutrition should aim to reduce advanced glycation end products (AGEs), which promote inflammation [69]. Although not SCI-specific, AGEs have been linked to degenerative cervical myelopathy [70]. Foods rich in melatonin or L-tryptophan (e.g., potatoes and chickpeas) possess anti-inflammatory properties and should be included [71, 72]. L-tryptophan aids serotonin and melatonin production and generates anti-inflammatory metabolites, such as indole-3-propionic acid via gut microbiota metabolism [73]. Similarly, L-arginine supports endothelial function, cardiovascular health, wound healing, and reduces inflammation [74].

#### Emerging perspectives on gut microbiome modulation in SCI

Recent studies link intestinal dysbiosis, in particular bacterial lipopolysaccharides (LPS), to SCI-related gastrointestinal dysfunctions, systemic low-grade inflammation, obesity, insulin resistance, diabetes, and cardiovascular disease [75, 76]. In SCI, LPS translocation worsens inflammation [77, 78], making intestinal barrier integrity crucial for preventing immune dysregulation and systemic complications [79]. Indeed, gut health and immunity are closely interconnected, and a balanced gut microbiome supports the production of short-chain fatty acids (SCFAs), which exert anti-inflammatory effects and regulate insulin [80]. Reduced microbial diversity, common in SCI, increases susceptibility to infections, such as *Clostridium difficile* [81] and multidrug-resistant *Staphylococcus aureus* (MRSA) [82]. Enhancing microbial diversity and SCFA levels involves greater intake of fruits, vegetables, fish, and dietary fibers, such as pectins, inulin and resistant starches [77, 83, 84]. Melatonin has also been shown to correct SCI-induced microbiota imbalances [85]. Specific strains, such as *Lactobacillus* and *Bifidobacterium* produce neuroactive compounds, such as gamma-aminobutyric acid (GABA). Those molecules influence both central and peripheral nervous systems and potentially reduce anxiety and depression symptoms [86].

#### Anti-inflammatory dietary components for enhancing immune function and gut health

The authors emphasized the importance of dietary modifications to harness the nutraceutical properties of food. Anti-inflammatory diets prioritize foods known to reduce chronic low-grade inflammation, a key driver of many non-communicable diseases. These dietary patterns emphasize fruits, vegetables, whole grains, legumes, nuts, seeds, olive oil, herbs and spices, and sources of omega-3 fatty acids, such as fish. Conversely, they limit red and processed meat, refined carbohydrates, added sugars and saturated fats, which are linked to chronic low-grade inflammation [87]. Such diets are also rich in polyphenols, antioxidants, vitamins, minerals, fiber, and healthy fats, all of which modulate inflammatory pathways, by lowering pro-inflammatory cytokines and oxidative stress. Evidence suggests that anti-inflammatory diets, including the Mediterranean diet, can help reduce inflammation-related risk across a range of conditions, from obesity to cancer and cognitive decline, by improving metabolic health, supporting gut microbiota, and dampening the molecular signals that sustain chronic inflammation [88].

Studies show that olive oil-based diets improve PI healing by reducing inflammation, oxidative stress, and enhancing collagen synthesis [89]. Anti-inflammatory diets have proven beneficial for SCI patients [90].

An optimal SCI diet should: 1) reduce inflammation and oxidative stress, 2) enhance immunity, 3) support gut microbiota, and 4) limit AGEs [69, 91]. Key features of such a diet include: 1) high omega-3 content [92], 2) low omega-6/omega-3 ratio [93], 3) high intake of polyphenols and antioxidants from herbs, fruits, and vegetables [94], 4) low glycemic load [95], and 5) minimal ultra-processed foods [96]. While direct links with SCI are unconfirmed, AGEs trigger oxidative stress and inflammation, impair endothelial function and angiogenesis, common issues in chronic illness [97]. Both food content and cooking methods affect AGE levels [98]. Strategies to reduce dietary AGEs include: 1) avoiding high-AGE foods, 2) using cooking methods that limit AGE formation, and 3) reducing intake of AGE-promoting ingredients, such as fructose. While foods rich in AGEs are aged and high-fat cheeses and dry-processed carbohydrates (e.g., crackers), AGE content increases depending on the cooking method (Table 4) [98, 99].

**Table 4:** Examples to Better Understand How the Cooking Method Can Be Decisive

Food and cooking method	AGE (kU/100 g)
Beef hot dog, boiled in water	7,484
Beef hot dog, grilled	11,270
Ground beef, pan-fried, previously marinated with lemon	3,833
Ground beef, pan-fried	4,928
Raw beef	707
Roast beef	6,071
Beef steak, grilled	7,479
Beef stew	2,657
Chicken breast, boiled in water	1,210
Chicken breast, breaded and fried	9,722
Chicken breast, steamed in aluminum foil	1,058
Chicken breast with skin, roasted	6,639
Chicken thigh with skin, roasted	10,997
Salmon, steamed	1,212
Salmon, roasted	4,334
Salmon fillet, boiled	1,082
Salmon fillet, grilled	3,347
Raw salmon	528
Smoked salmon	572
Cheese, light ricotta (1% fat)	1,453
Cheese, feta	8,423
Cheese, Parmesan	16,900
Egg, omelet, low heat with cooking spray, 11 min	90
Egg, omelet, low heat with butter, 13 min	507
Egg, omelet, low heat with olive oil, 12 min	337

AGE: advanced glycation end product.

Anti-inflammatory compounds, such as quercetin, resveratrol, curcumin, and catechin, can help counteract AGE accumulation [100].

Hydration is essential for wound healing and skin health. Recommended fluid intake is 1 mL per kcal of energy needs plus 500 mL, or 40 mL/kg of body mass plus 500 mL [101]. Additional fluids are needed for those with draining wounds or excessive sweating. Immunonutrition formulas, typically including arginine, glutamine, omega-3s, vitamins, and trace minerals, have been associated with reduced infections and inflammation and improved gut health and wound healing [102]. These dietary principles form the foundation of nutritional strategies for SCI patients with or at risk for PIs (Table 5).

**Table 5:** Key Dietary Components to Improve Immune Function and Gut Health

Fruits, vegetables, and resistant starches (providing soluble and insoluble fibers). Fish, which contributes to greater microbiota biodiversity. Foods rich in tryptophan and arginine are essential for metabolic and immune functions. Fermented foods provide beneficial probiotics.

**Figure 2:**
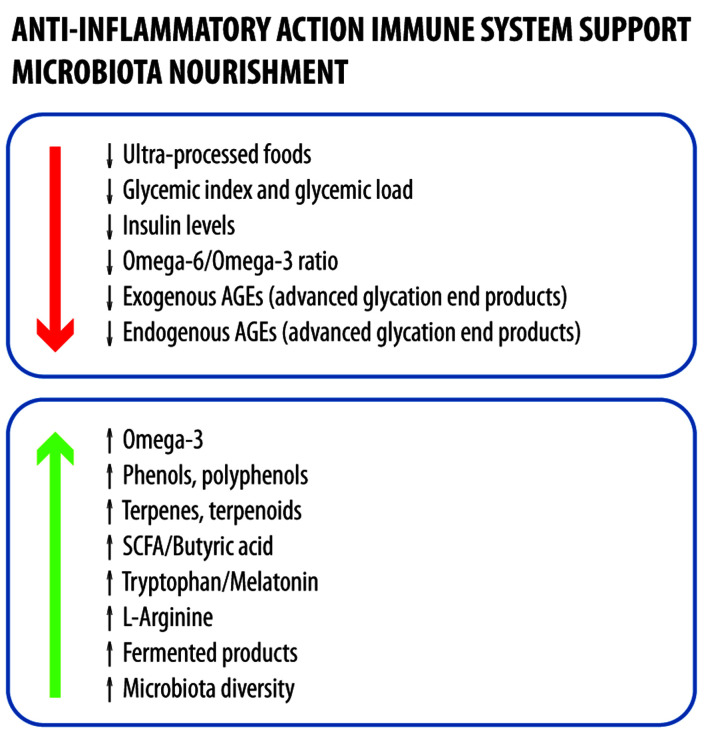
Characteristics of an anti-inflammatory diet that promotes gut health and limits the amount of advanced glycation end products.

Patient education on precise dietary choices is essential and should be viewed as part of standard medical care. The proposed dietary plan is based on Mediterranean diet, which is consistently showing beneficial effects on chronic conditions [103], and targets an 81-kg tetraplegic patient with no major comorbidities (Tables 6–11). The plan assumes an energy intake of ∼20 kcal/kg body weight and includes 2 L of calcium-rich water (≥ 300 mg/L). Key points include: 1) sugar intake only from whole foods; 2) optimal calcium intake, excluding water contribution; 3) adequate intake of vitamins, minerals, and amino acids; and 4) suboptimal resveratrol levels, which require supplementation (Fig. 2).

**Table 6:** Breakfast With Whole Oat Porridge With Berries, Flaxseeds, Kefir, and Almonds

Ingredients	Nutritional values
40 g whole oats 100 g berries 5 g flaxseeds 150 mL kefir 3 almonds	Energy: 375 kcal Carbohydrates: 52 g Proteins: 13 g Fats: 11 g Saturated fats: 1.8 g Fiber: 8.0 g Simple sugars: 10 g Vitamin A: 110 IU Vitamin C: 25 mg Vitamin B12: 0.5 µg Quercetin: 7 mg Tryptophan: 180 mg Melatonin: 0.7 µg Inulin: 1.5 g Omega-6: 1.7 g Omega-3: 1.2 g Omega-6/omega-3 ratio: 1.4:1 Arginine: 0.5 g Glutamine: 1 g Zinc: 1.1 mg Calcium: 180 mg Glycemic load: 14 Curcuminoids: 0 mg Gingerol: 0 mg Piperine: 0 mg Polyphenols: 70 mg Resveratrol: 0.5 mg

**Table 7:** Snack With Pear, Almonds, and Green Tea

Ingredients	Nutritional values
150 g pear 10 g almonds 1 cup green tea	Energy: 150 kcal Carbohydrates: 20 g Proteins: 3.0 g Fats: 8.0 g Saturated fats: 0.6 g Fiber: 4 g Simple sugars: 14 g Vitamin A: 5 IU Vitamin C: 7 mg Vitamin B12: 0 µg Quercetin: 0.3 mg Tryptophan: 35 mg Melatonin: 0.1 µg Inulin: 0 g Omega-6: 1.7 g Omega-3: 0.01 g Omega-6/omega-3 ratio: 170:1 Arginine: 0.4 g Glutamine: 0.3 g Zinc: 0.6 mg Calcium: 20 mg Glycemic load: 8 Curcuminoids: 0 mg Gingerol: 0 mg Piperine: 0 mg Polyphenols: 40 mg Resveratrol: 0 mg

**Table 8:** Lunch With Whole Wheat Pasta With Chickpeas, Artichokes, Red Onion, Chicory; Steamed or Boiled Chicken Breast; Salad of Fresh Spinach, Red Onion, Avocado, Olive Oil, Turmeric, Ginger, and Black Pepper

Ingredients	Nutritional values
80 g whole wheat pasta 50 g dried chickpeas (rehydrated) 50 g artichokes 50 g chicory 100 g chicken breast 50 g red onion 100 g fresh spinach 30 g avocado 5 g olive oil 5 g turmeric, ginger, and black pepper	Energy: 515 kcal Carbohydrates: 90 g Proteins: 35 g Fats: 10 g Saturated fats: 2.5 g Fiber: 14 g Simple sugars: 7 g Vitamin A: 9,733 IU Vitamin C: 47 mg Vitamin B12: 0.2 µg Quercetin: 20 mg Tryptophan: 320 mg Melatonin: 0.4 µg Inulin: 5 g Omega-6: 3.8 g Omega-3: 1.2 g Omega-6/omega-3 ratio: 3.2:1 Arginine: 1.8 g Glutamine: 3.0 g Zinc: 2.3 mg Calcium: 130 mg Glycemic load: 23 Curcuminoids: 150 mg Gingerol: 20 mg Piperine: 5 mg Polyphenols: 100 mg Resveratrol: 0.01 mg

**Table 9:** Snack With Greek Yogurt With Chia Seeds and Green Tea

Ingredients	Nutritional values
150 g Greek yogurt 10 g chia seeds 1 cup green tea	Energy: 149.5 kcal Carbohydrates: 9.0 g Proteins: 11.0 g Fats: 8.0 g Saturated fats: 3.0 g Fiber: 3.0 g Simple sugars: 5 g Vitamin A: 40 IU Vitamin C: 0 mg Vitamin B12: 0.6 µg Quercetin: 0 mg Tryptophan: 90 mg Melatonin: 0 µg Inulin: 0 g Omega-6: 1.5 g Omega-3: 0.5 g Omega-6/omega-3 ratio: 3:1 Arginine: 0.8 g Glutamine: 1.2 g Zinc: 1.2 mg Calcium: 140 mg Glycemic load: 5 Curcuminoids: 0 mg Gingerol: 0 mg Piperine: 0 mg Polyphenols: 60 mg Resveratrol: 0 mg

**Table 10:** Dinner With Baked Salmon Fillet With Broccoli, Red Onion, Whole-Grain Bread, Olive Oil, Turmeric, Ginger, and Black Pepper

Ingredients	Nutritional information
Salmon fillet 150 g Broccoli 150 g Red onion 50 g Whole-grain bread 50 g Olive oil 5 g Turmeric, ginger, black pepper 5 g	Energy: 515 kcal Carbohydrates: 45 g Proteins: 37 g Fats: 22 g Saturated fats: 4.4 g Fiber: 10 g Simple sugars: 5 g Vitamin A: 617 IU Vitamin C: 86 mg Vitamin B12: 4.5 µg Quercetin: 13 mg Tryptophan: 325 mg Melatonin: 0.3 µg Inulin: 1.5 g Omega-6: 3 g Omega-3: 2.5 g Omega-6/omega-3 ratio: 1.2:1 Arginine: 1.9 g Glutamine: 2.7 g Zinc: 2.8 mg Calcium: 160 mg Glycemic load: 12 Curcuminoids: 150 mg Gingerol: 20 mg Piperine: 5 mg Polyphenols: 120 mg Resveratrol: 0 mg

**Table 11:** Total Daily Intakes

Nutritional components	Nutritional information
Energy	1,695 kcal
Carbohydrates	213 g (50%)
Proteins	97.7 g (23%)
Fats	58 g (27%)
Saturated fats	12.1 g (6%)
Fiber	49 g
Simple sugars	41 g
Vitamin A	10,505 IU
Vitamin C	165 mg
Vitamin B12	5.8 µg
Quercetin	40.3 mg
Tryptophan	960 mg
Melatonin	1.5 µg
Inulin	8.0 g
Omega-6	11.2 g
Omega-3	6.3 g
Omega-6/omega-3 ratio	1.8:1
Arginine	5.1 g
Glutamine	7.8 g
Zinc	7.9 mg
Calcium	620 mg
Glycemic load	61
Curcuminoids	300 mg
Gingerol	40 mg
Piperine	10 mg
Polyphenols	390 mg
Resveratrol	0.06 mg

### Patient-setting specific management strategies

Effective PI management includes enhancing psychosocial support, ensuring complete pressure relief, optimizing support surfaces and wheelchair seating systems, and providing adequate nutrition. In addition, reducing wound bioburden, eradicating infections, and, when necessary, performing surgical interventions followed by appropriate postoperative care all form essential components of the overall management strategy [104].

#### Management strategies for the “acute” setting: in-hospital care

In the acute setting, verifying functional outcomes and using pressure redistribution devices are essential [104]. Physical activity helps prevent muscle atrophy, PIs, obesity, inactivity, and bone loss [105]. Patient weight should be assessed to ensure devices fit properly, as standard models may not suit individuals with obesity. For positioning and pressure relief [106], repositioning is advised every 2–4 h with support from nurses or therapists. Wheelchair users should shift weight for at least 30 s every 15 min and reposition hourly [13]. Nutritional support within 72 h is standard for trauma patients. Ongoing clinical and instrumental assessments are needed during acute and post-acute phases, with interventions tailored to high-risk individuals [59]. At discharge, nutrition counseling and assessing awareness of the role of diet in PI prevention and healing are strongly recommended.

#### Management strategies for the “chronic” setting

**Figure 3:**
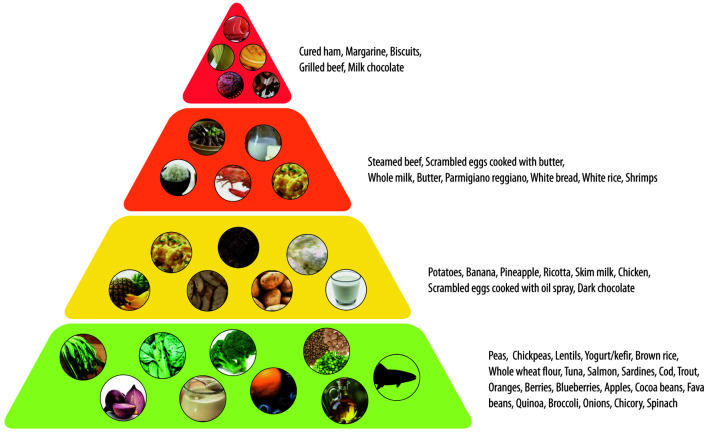
A traffic light food map for SCI patients with PIs. PIs: pressure injuries; SCI: spinal cord injury.

In the chronic setting, PI prevention strategies mirror those in acute and post-acute phases and include daily skin checks for early detection, minimizing risk factors, and maintaining a balanced diet with nutritional counseling [107]. The authors emphasized the importance of educating patients and caregivers on PI risk factors and the role of nutrition in skin integrity and healing. To simplify dietary guidance, the authors introduced a Food Suitability Map that uses color-coding to classify foods based on nutritional value, drawing from international literature and databases. Foods are categorized by intake frequency within an anti-inflammatory Mediterranean diet [88, 103, 108]. Foods classified as “green” are those for daily use, with high therapeutic value. Foods classified as “yellow” are those for moderate weekly use with limited benefits. Foods classified as “orange” are those for occasional weekly use and display minimal benefit or mild risk. Foods classified as “red” are those for rare intake or to be avoided, due to potential harm and limited value (Fig. 3).

Additional factors, such as bowel and sphincter function, colostomy presence, diabetes, QoL, physical activity, and eating habits, should be evaluated, with a 1-month follow-up recommended.

**Figure 4:**
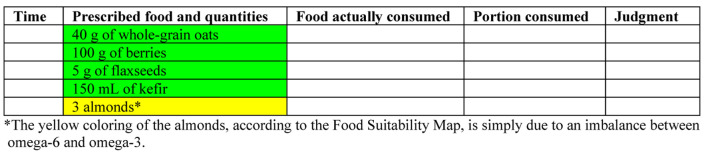
Template of a food diary for recording consumption according to the prescribed meal plan for breakfast.

**Figure 5:**
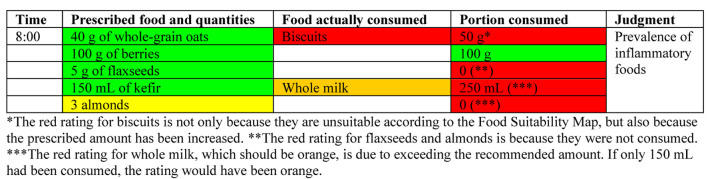
Example of how a food diary could look after patient interaction.

A food diary is essential for SCI patients to track intake, support metabolic health, PI prevention, and adherence to personalized plans. As biomarkers provide limited insight due to physiological variability and cannot guide dietary change [109], direct food tracking remains the most practical method. The authors advocate for a food diary as an interactive tool involving patients and caregivers, enhanced by the Food Suitability Map. This integration helps identify dietary patterns and necessary modifications. Core features of an effective food diary are listed in Table 12, and the diary templates (Figs 4 and 5) align with the Nutritional Therapeutic Plan. Color-coded entries reflect prescribed meals and guide appropriate substitutions based on the Suitability Map. A sample entry is shown in Figure 5.

**Table 12:** Key Features of a Food Diary for Spinal Cord Injury Patients

It should outline the patient’s dietary tasks (diet). It must allow for the study, implementation, and recording of alternatives to the prescribed dietary recommendations. It must allow a final overall assessment through the Food Suitability Map, based on the prevalence of strongly inflammatory or, conversely, health-beneficial foods. It should not be a mere summary of quantities and energy intake but a tool to help the patient truly perceive what has the greatest impact on their health.

#### Management strategies for the “chronic managed by local healthcare services” setting

The authors highlighted the need for professional figures in this setting, as certified specialists in SCI management are often lacking. To address this, the authors suggested periodic follow-ups and specialized training programs to enable HCPs to provide optimal care. The recommendations outlined for other care settings should also be applied in this context.

#### Management strategies for the “chronic patients readmitted to the hospital with complications” setting

A concerning statistic regards hospital readmissions of chronic SCI, 40% of cases of which are due to PIs. The authors emphasized the need for effective community-level handovers, as spinal units alone cannot meet growing care demands. A structured network between hospitals and local HCPs, based on clear communication, defined roles, and coordinated actions, is crucial for ensuring care continuity. In PI surgery, nutritional interventions should be initiated not only postoperatively [110], but also preoperatively as part of a “prehabilitation” strategy. The authors support incorporating dietary measures into a tailored Enhanced Recovery After Surgery (ERAS) protocol, encompassing pre-, intra-, and postoperative phases to improve recovery, prevent complications, and optimize outcomes [104]. Before PI-related surgery, patients should undergo gastroenterological evaluation to assess colostomy needs. Rehabilitation specialists and occupational therapists (OTs) evaluate home care, seating, pressure relief, and bladder function. Dietitians help tailor nutrition plans to support wound healing and muscle maintenance. OTs also assess skin integrity, identify risks, recommend preventive strategies, evaluate the adequacy of pressure-relief devices, and guide patients and caregivers on skin inspection. Psychologists support emotional well-being and adherence to care plans, enhancing motivation, coping, and long-term self-management [13, 111].

## Conclusions

Managing patients with SCIs requires a multidisciplinary approach, with nutrition playing a key role in preventing and treating PIs. Individualized dietary strategies are essential to address SCI-related metabolic and inflammatory issues. Supporting gut health with fiber, probiotics, and anti-inflammatory foods alongside the intake of micronutrients (e.g., vitamins A, C, zinc, iron) and adequate hydration can promote wound healing and lower PI risk, helping maintain immune function and reduce inflammation. Personalized dietary plans, guided by qualified dietitians, should meet energy and nutrient needs in SCI. Tools such as food diaries and dietary maps can enhance education, adherence, and informed decision-making. Implementing evidence-based nutrition protocols can improve outcomes, reduce costs, and enhance QoL for SCI patients. Future research should refine dietary interventions, evaluate nutraceuticals, and assess the long-term impact of nutrition on PI prevention and healing.

## Data Availability

The authors declare that data supporting the findings of this study are available within the article.
